# Chimeric antigen receptor T cells self-neutralizing IL6 storm in patients with hematologic malignancy

**DOI:** 10.1038/s41421-021-00299-6

**Published:** 2021-09-14

**Authors:** Lei Xue, Yan Yi, Qianwen Xu, Li Wang, Xiaohui Yang, Yongjing Zhang, Xuefei Hua, Xiaoshan Chai, Junjie Yang, Yaxin Chen, Guangshi Tao, Biliang Hu, Xingbing Wang

**Affiliations:** 1 The First Affiliated Hospital of University of Science and Technology of China (USTC), Hefei, Anhui China; 2 The Second Xiangya Hospital of Central South University, Changsha, Hunan China; 3 Hunan Key Laboratory of Tumor Models and Individualized Medicine, Changsha, Hunan China; 4 Celledit, Worcester, MA USA; 5 Siweikang Therapeutics, Changsha, Hunan China

**Keywords:** Cancer therapy, Cell biology

## Abstract

IL6 is one of the most elevated cytokines during chimeric antigen receptor (CAR) T cell cytokine release syndrome (CRS), and IL6R blockade by Tocilizumab has successfully relieved the most life-threatening aspects of CRS in patients. In addition, latest studies demonstrated the essential role of IL1 in driving CART induced neurotoxicity in mouse models. Here we present a clinical investigation (ChiCTR2000032124; ChiCTR2000031868) of anti-CD19 and anti-BCMA CART (41BBζ) secreting an anti-IL6 scFv and IL1 receptor antagonist (IL1RA) in treating patients with hematologic malignancy. Our results revealed that IL6 and IL1B were maintained at low levels without significant elevation during CRS, rendering Tocilizumab dispensable. Moreover, treated patients did not show neurotoxicity during CRS and exhibited mild to moderate CRS. Notably, we observed high rate of complete response (CR) and significant CART expansion during treatment. In sum, we conclude that CART-secreting anti-IL6 scFv and IL1RA could self-neutralize IL6 storm and maintain low levels of IL1B during CART therapy to minimize IL6- and IL1-associated cytokine toxicity and neurotoxicity without impairing therapeutic efficacy.

## Introduction

Although chimeric antigen receptor (CAR) T therapy has demonstrated breakthrough efficacy in hematologic malignancies^1^, life-threatening cytokine release syndrome (CRS) and neurotoxicity remain as one of the major challenges in the clinical practice of CART treatment^2^. The rate of severe CRS (grade 3 or greater) for Tisagenlecleucel was 23% in refractory/relapsed B-cell lymphoma^3^ and 46% in acute lymphoblastic leukemia (ALL)^4^. The rate of severe neurotoxicity (grade 3 or greater) was 30% for axicabtagene ciloleucel in refractory/relapsed B-cell lymphoma^5^, 21% for anti-CD19 CART clinic trial in ALL led by Fred Hutchinson Cancer Research Center (FHCRC)^6^, and 42% for anti-CD19 CART clinic trial in ALL led by Memorial Sloan Kettering Cancer Center (MSKCC)^7^. Moreover, there were 2 (out of 93) fatal cases (encephalitis and cerebral hemorrhage) for Tisagenlecleucel in lymphoma^3^, 3 (out of 108) fatal cases (cardiac arrests; HLH; pulmonary embolus) for axicabtagene ciloleucel in lymphoma^5^, and 2 (out of 75) fatal cases of cerebral hemorrhages for Tisagenlecleucel in ALL^4^. In addition, a previous clinical trial of a CAR with a CD28 costimulatory signaling in adults with ALL was terminated after five cases of fatal cerebral edema^8^.

The severity of CRS depends on multiple parameters, including but not limited to tumor burden, CART proliferation, and inflammation of patient’s immune system during CART-mediated eradication of tumor cells. Since these parameters are highly variable from patient to patient, it is very difficult to accurately predict which patient should be given early intervention for preventing severe CRS and neurotoxicity. Moreover, severe CRS may lead to additional dysfunctions in different organs, such as heart, liver, lung, and kidney^9^. Meanwhile, it should be taken into consideration that patients in late stage with refactory and relapsed hematologic malignancy usually have received multiple standard treatments, which may dampen patients’ health capacity to survive through severe CRS. Currently, there is no clinically approved CART therapy capable of preventing CRS and neurotoxicity, therefore requiring close monitoring of patients to alleviate severe CRS and neurotoxicity by Tcilizumab and corticosteroids and supportive treatment for CRS-associated complications in order to avoid fatal adverse events. Taken together, it is urgent to explore novel design of CART therapy for automatically reducing CRS and neurotoxicity.

So far, there have been several strategies investigated for evolving CAR designs to resolve CRS. Inducible Caspase9^10–12^ has also been incorporated in CART cells (NCT02414269), in which inducer drug AP1903 can activate Caspase9 dimerization to elicit CART apoptosis during CRS. As well, surface labeling of CART cells by CD20^13^ or tEGFR^14^ have been proposed to eliminate CART cells by CD20- or tEGFR-targeted antibodies during severe CRS (NCT03618381; NCT03085173). However, these strategies rely on elimination of CART cells to reduce CRS, which will simultaneously impair therapeutic efficacy. Moreover, manual interference with AP1903 or CD20/tEGFR targeted antibodies will take certain time to effectively eliminate CART cells, which may not be able to provide immediate rescue to patients undergoing severe CRS.

IL6 is one of the most significantly elevated cytokines during CRS^15^ and becomes the focus of clinical interest in CRS management, as IL6 signaling blockade by Tocilizumab (targeting IL6R) has relieved the most life-threatening aspects of CRS in patients^16^. IL6 trans-signaling has been suggested to contribute to many characteristic symptoms of severe CRS, such as vascular leakage, and activation of the complement and coagulation cascade inducing disseminated intravascular coagulation (DIC)^17^. Since IL6 does not play an essential role for the efficacy of CART therapy^16^, Tocilizumab has been approved by FDA to treat severe CRS^18^. However, one of the disadvantages of Tocilizumab might be that binding to IL6R still allows high level of serum IL6 to circulate and potentially cross blood-brain barrier (BBB), which might cause severe neurotoxicity. As well, there is lack of quantitative standard when and how much Tocilizumab should be used in clinical practice, which becomes really challenging especially when serum IL6 increases dramatically in very short time during CART therapy. Lastly, Tocilizumab administration adds significant medical cost on top of the highly expensive CART therapy^19^. In addition to IL6, two independent studies had elaborated the essential and differential roles of IL6 and IL1 in causing severe CRS and neurotoxicity in mouse models, and CART has been designed to secrete IL1RA for preventing CRS^20,21^. Taken together, IL6 and IL1 serve as promising targets for resolving severe CRS and neurotoxicity in CART therapy.

During severe CRS, significant elevation of IL6 secreted by endogenous macrophages^16^ is usually occurring at peak expansion of CART cells. Therefore, we hypothesized that highly proliferating CART cells serve as an ideal vehicle of automatically synthesizing large amount of cytokine antagonists against IL6 and IL1 during CRS. To this aim, we engineered anti-CD19 or anti-BCMA (B cell maturation antigen) CART to autonomously co-express an anti-IL6 single-chain variable fragment (scFv) (aIL6) for blocking IL6 signaling and IL1RA for blocking IL1 signaling, referred as CART-aIL6/IL1RA. Patients with refractory/relapsed ALL or chronic lymphoblastic leukemia (CLL), lymphoma, and multiple myeloma (MM) were enrolled. In this study, we present the initial clinical results on the effect of CART-secreted aIL6 and IL1RA for reducing IL6- and IL1-associated cytokine toxicity and neurotoxicity.

## Results

### Design of CART secreting aIL6 scFv and IL1RA

**Fig. 1:**
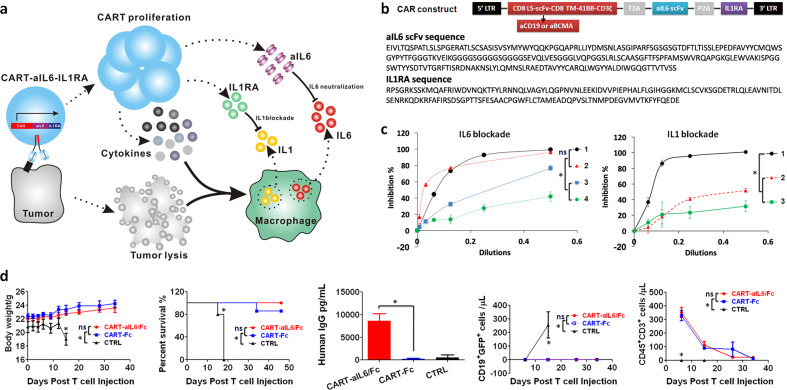
Design of CART for secreting IL6 and IL1 antagonists. **a** The schematic illustration of CART-secreted anti-IL6 scFv and IL1RA in blocking IL6 and IL1 signalings during CRS of CART therapy. **b** The design of CAR construct and the sequences of aIL6 scFv and IL1RA. **c** Comparison of gene delivered expression of different scFv targeting IL6 or IL6R in blocking IL6 signaling and the scFv was consisting of variable regions derived from 1-Sarilumab, 2-Sirukumab, 3-Siltuximab or 4-Tocilizumab. Optimization of co-expressing IL1RA with aIL6 for inhibiting IL1 signaling. Construct 1 includes, from N-terminus to C-terminus, T2A linker, scFv derived from Sirukumab, P2A linker, and IL-1RA (T2A-Sir-P2A-IL1RA). Construct 2 contains, from N-terminus to C-terminus, T2A linker, scFv antibody, (G4S)3 linker, and IL1RA (T2A-Sir-(G4S)3-IL1RA). Construct 3 contains, from N-terminus to C-terminus, scFv antibody, (G4S)3 linker, IL1RA, and T2A linker (Sir-(G4S)3-IL1RA-T2A). Triplicate was included in the test. **d** Anti-tumor efficacy of anti-CD19 CART-aIL6/Fc (*n* = 8) and CART-Fc (*n* = 7) in Xenograft model and quantification of CART-secreted aIL6/Fc in D6 serum. The representative results of two independent experiments were presented. Student’s *t*-test was conducted, and * denotes statistical significance of *P* < 0.05. ns means no significance.

By taking advantage of highly proliferating CART cells during CRS, we designed CART to autonomously secrete anti-IL6 scFv and IL1RA for blocking IL6 and IL1 signalings during CART therapy as illustrated in Fig. 1a, b. First of all, we demonstrated that the 3rd generation lentivector was successfully engineered to deliver anti-IL6 scFv derived from Sirukumab and IL1RA in efficiently inhibiting IL6 and IL1 signalings in vitro (Fig. 1c). Although it was comparable in inhibiting IL6 signaling in vitro between scFvs derived from Sarilumab and Sirukumab, we chose the scFv derived from Sirukumab for the following study because it is targeting IL6 instead of IL6R. Next, to explore whether the incorporation of aIL6 scFv would affect the anti-tumor activity of CART cells, we inoculated NCG mice with GFP-expressing Nalm6 cells and treated them with anti-CD19 CART-aIL6/Fc, which demonstrated efficient eradication of GFP^+^ tumor cells and long term survival as compared to anti-CD19 CART/Fc (Fig. 1d). Furthermore, quantification of aIL6-Fc by enzyme-linked immunosorbent assay (ELISA) showed that CART could efficiently synthesize and secrete aIL6 scFv for binding to IL6 (Fig. 1d). In addition, CD45^+^CD3^+^ human T cells decreased gradually after tumor eradication without body weight loss after infusion (Fig. 1d), suggesting that the anti-CD19 CART-aIL6/Fc CART cells were effective and safe in the mouse model.

### CART-aIL6/IL1RA mediated self-neutralization of IL6 storm in treated patients

**Fig. 2:**
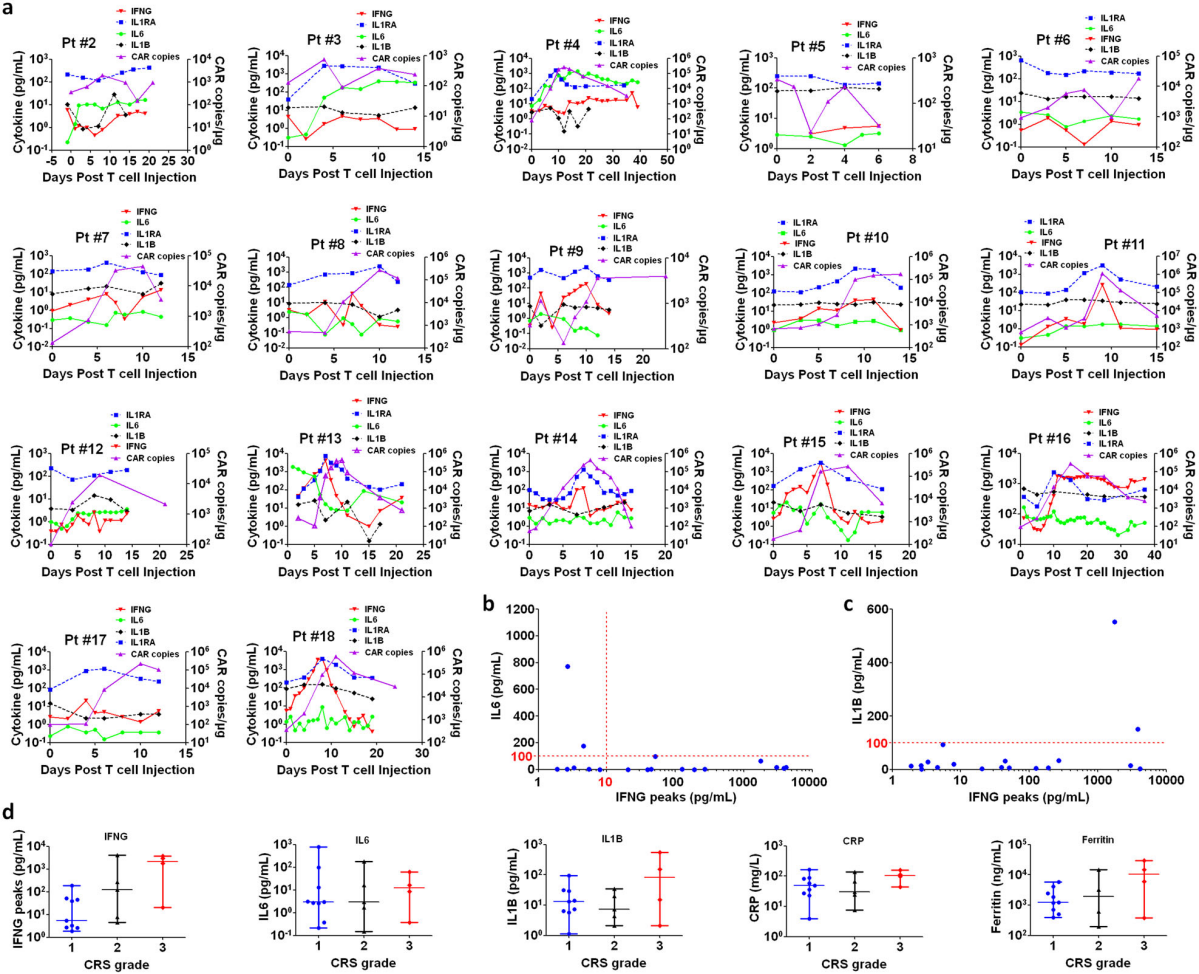
CART expansion, cytokine changes, and CRS in the patients. Patients (as numbered in Table 1) with refractory or relapsed ALL, CLL, Lymphoma, or MM were enrolled and treated with anti-CD19 or anti-BCMA CART secreting anti-IL6 scFv and IL1RA. After CART infusion, patients were monitored for clinical signs of CRS and examined for clinical response of tumor remission. **a** Changes of IFNG, IL6, IL1RA, IL1B, and CAR vector copies in blood. **b** Plot of IFNG peak levels versus the IL6 concentration at the time of IFNG peak during CRS. **c** Plot of IFNG peak levels versus the IL1B concentration at the time of IFNG peak during CRS. **d** The relationship between CRS gradings and IFNG peaks, or IL6, IL1B, CRP, and Ferritin levels at the time of IFNG peaks during CRS.

Having demonstrated the capacity of CART cells co-secreting IL6 and IL1 blockers and their safety in mouse model, we initiated a clinical investigation of the safety and efficacy of CART-aIL6/IL1RA for targeting CD19 or BCMA, with 18 patients enrolled, including ALL, CLL, lymphoma and multiple myeloma (MM) as summarized in Table 1. After CART infusion, CART vector copies and changes of cytokines were monitored, including IFNG, IL6, IL1B, and IL1RA. As shown in Fig. 2a, IL1RA levels were positively correlated with the kinetics of CART expansion and IFNG secretion. It is of significance to note that IL1RA elevated significantly as the CART expanded and decreased to low levels close to baseline as CART copies decreased, suggesting that CART-mediated secretion of aIL6 scFv and IL1RA was transiently active while CART was killing target cells and proliferating. After CART cells rested, the observation that CART-mediated secretion of IL1RA decreased to baseline levels significantly relieved the safety concern of interfering with patient endogenous immune system after treatment was completed.

**Table 1:** Summary of patients treated with CART-aIL6/IL1RA.

Pt #	Gender	Age (yrs)	Dose (×10^8^)	IFNG (pg/mL)	CRP (mg/L)	Ferritin (ng/mL)	IL1B (pg/mL)	IL6 (pg/mL)	Response	Disease	CRS	Neurotoxicity (during CRS)	Tocilizumab
1	F	66	0.4	50.85	166	4052.8	6.32	97.41	CR	MM	1	Yes^a^	No
2	M	57	0.15	3.33	3.94	497.74	28.74	12.93	PR	MCL	1	None	No
3	M	53	0.2	4.54	24.8	608.99	7.31	177.01	PR	MCL	2	None	No
4	M	53	0.2	2.69	22.6	1872.79	1.15	772.01	CR	MM	1	None	No
5	F	6	0.5	5.48	27.5	706.5	93	3.15	CR	ALL	1	None	No
6	M	24	0.4	1.88	35.8	1176	13.03	2.65	NR	ALL	1	None	No
7	F	17	0.3	7.9	7.57	195.4	20.27	0.15	CR	ALL	2	None	No
8	M	26	0.1	39.18	81.5	2432.6	7.31	0.37	CR	ALL	1	None	No
9	M	57	0.2	188.37	57.5	1223.7	5.82	0.22	CR	MCL	1	None	No
10	F	6	0.3	44.42	89.3	5828	31.71	2.95	CR	ALL	1	None	No
11	M	24	0.4	266.63	138	15000	34.22	1.71	CR	ALL	2	None	No
12	M	38	0.25	2.63	49.8	391.9	14.29	2.71	NR	TCRBCL	1	None	No
13	F	49	1.3	4118.03	30.1	NA	2.15	16.36	CR	ALL	2	None	No
14	F	21	0.35	124.89	66.3	3247	4.32	2.93	CR	ALL	2	None	No
15	F	39	0.5	2979.29	157	6029.1	15.29	16.34	CR	ALL	3	None	No
16	M	47	0.45	1751.76	99.1	15000	552.34	63.02	PR	CLL	3	None	No
17	F	52	0.2	20.22	43.9	386.6	2.15	0.37	CR	MCL	3	None	No
18	F	46	0.3	3812.54	113	30000	151.29	8.56	CR	ALL	3	None	No

*CR* complete response, *PR* partial response, *NR* no response, *ALL* acute lymphoblastic leukemia, *CLL* chronic lymphoblastic leukemia, *MCL* mantle cell lymphoma, *TCRBCL* T-cell/histiocyte-rich large B-cell lymphoma, *MM* multiple myeloma.

CRS period was defined as the time window when patient was showing active CART proliferation, IFNG secretion and/or fever.

^a^No neurotoxicity observed during CRS, and neurotoxicity present after CRS ended. IFNG value denotes the peak level during CRS, and IL6, IL1B, CRP, and Ferritin values denote the concentrations at the time of IFNG peaks.

During traditional CART therapy, IFNG is the major indicator reflecting the level of CART expansion and cytotoxicity against tumor cells, and significantly elevated IFNG overlaps closely with highly increased IL6^15^. In sharp contrast, 16 out of the 18 patients (Fig. 2a, b) treated with CART-aIL6/IL1RA consistently displayed low levels of serum IL6 (< 100 pg/mL) during CRS, whereas the IFNG peaks varied a lot from very low levels to extremely high levels (ranging from 2.63 to 4118.03 pg/mL). Notably in 7 out of these 15 patients, while IFNG significantly increased to more than 100 pg/mL, there was no significant elevation of IL6 observed (Fig. 2b). Only 2 out of these 18 patients showed moderate increase of IL6 (177.01 and 772.01 pg/mL) during Grade 1 CRS (fever), probably due to extremely low levels of CART activity as reflected by IFNG secretion. In addition, these 2 patients showed further increase of IL6 (390.22 and 1404 pg/mL) after Grade 1 CRS (fever) ended, but did not cause any further toxicity. In summarizing the results of all 18 patients (Fig. 2b), the IL6 levels at the time of IFNG peaks were plotted versus the corresponding IFNG peaks. These results provided clear evidence that highly proliferating CART were capable of secreting anti-IL6 scFv to self-neutralize IL6 storm during CRS.

### No significant elevation of serum IL1B was observed in CART-aIL6/IL1RA-treated patients

In the previous study, significantly elevated IL1B was observed in CART-treated patients^15^, suggesting the significance to block or reduce IL1 signaling during CRS. In the current study, our results proved that IL1RA significantly increased as CART expanded and serum IFNG elevated after infusion (Fig. 2a), suggesting that CART cells were capable of efficiently secreting IL1RA while killing target cells. Surprisingly, 16 out of 18 patients displayed low levels of IL1B during treatment (< 100 pg/mL) (Fig. 2a and c). Only patients #16 and #18 showed moderate levels of IL1B peaks (552.35 and 151.29 pg/mL), which did not increase significantly as compared to the baseline levels (695.83 and 87.53 pg/mL). Interestingly in these 2 patients, the levels of IL6 were all kept at low levels (< 100 pg/mL). These results suggested that CART-secreting aIL6 and IL1RA could efficiently restrain IL1B elevation during CART therapy.

### Automatic IL6 neutralization and IL1 blockade eliminated neurotoxicity during CRS

**Fig. 3:**
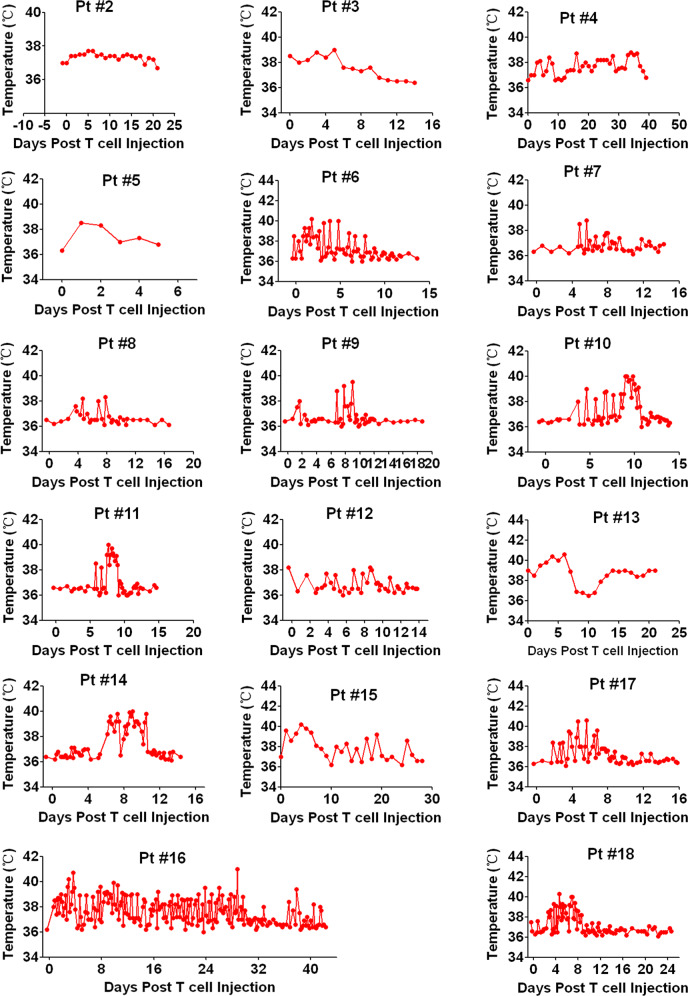
Changes of body temperature in patients treated with CART-aIL6/IL1RA. Patients (as numbered in Table 1) with refractory or relapsed ALL, CLL, Lymphoma, or MM were enrolled and treated with anti-CD19 or anti-BCMA CART secreting anti-IL6 scFv and IL1RA. After CART infusion, patients were monitored for recording body temperature to assess grading of CRS.

**Fig. 4:**
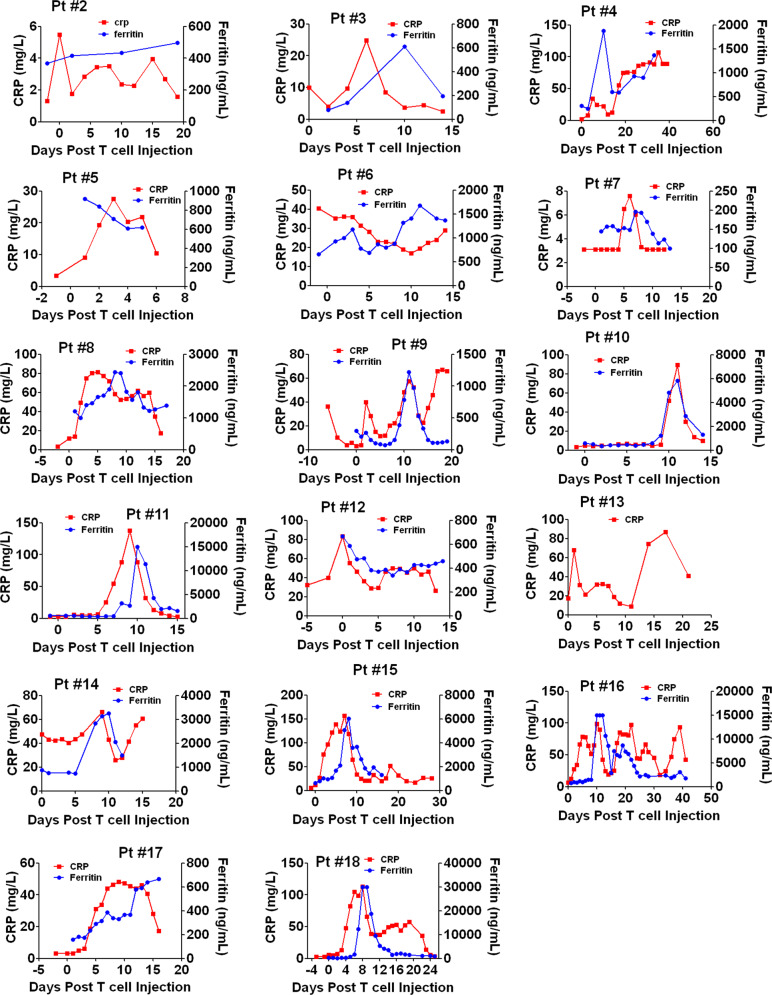
Changes of CRP and Ferritin in patients treated with CART-aIL6/IL1RA. Patients (as numbered in Table 1) with refractory or relapsed ALL, CLL, Lymphoma, or MM were enrolled and treated with anti-CD19 or anti-BCMA CART secreting anti-IL6 scFv and IL1RA. After CART infusion, patients were monitored for recording CRP and Ferritin levels to assess grading of CRS.

In the current study, CRS of each patient was graded according to previous ASTCT CRS Consensus Grading Criteria^22^. Among these 18 patients, 4 patients experienced grade 3 CRS, 5 patients experienced grade 2 and 9 patients experienced only grade 1 CRS, whereas none patient displayed neurotoxicity during CRS period (Table 1 and Fig. 2d). The changes of body temperatures were shown in Fig. 3. Except Pt #1 displayed neurotoxcity after CRS ended, the rest 17 patients did not exhibit neurotoxicity throughout the treatment. Among these 4 patients with grade 3 CRS, Pt #16 and #18 showed extremely high levels of Ferritin during CRS (Fig. 4). Pt #15 showed high level of IFNG peak, but low levels of IL1B and IL6 (Fig. 2a). Interestingly, Pt #17 showed low levels of IFNG, IL6, IL1B, C-reactive protein (CRP) and Ferritin (Fig. 2a), suggesting the grade 3 CRS might be attributed to tumor lysis syndrome or other inflammation pathways. Notably, none of these patients showed high levels of IL6 and IL1B simultaneously during CRS, which may explain the absence of neurotoxicity during CRS. These results suggested that CART-secreted aIL6 and IL1RA could effectively prevent significant elevation of IL6 and IL1B during CRS, therefore minimizing IL6- and IL1-associated cytokine toxicity and neurotoxicity.

### Tocilizumab was dispensable for treating CRS in CART-aIL6/IL1RA treated patients

Among the 18 patients, 16 patients displayed low levels of IL6 during CRS, and 2 patients displayed moderate levels of IL6 with very low levels of IFNG during only grade 1 CRS, therefore not requiring Tocilizumab for blocking IL6 signaling. Only Pt #1 showed neurotoxicity associated with high IL6 level after CRS ended without any other cytokines elevated, therefore treatment for IL6 blockade would be desirable. However, Tocilizumab is binding to IL6R and may not cross BBB due to its large size, and therefore it was not applied in this patient due to the concern of high level of serum IL6 to cross BBB for worsening neurotoxicity.

Among these 18 patients, only Pt #11, 16, and 18 received corticosteroids due to significant increase of Ferritin (Fig. 4), because high level of Ferritin was suggested to be specific and sensitive for hemophagocytic lymphohistiocytosis (HLH)^23^, persistent fever or cardiotoxicity. For Pt #11, significant elevation of Ferritin was observed and subsequently treated with relatively low doses of dexamethasone (20 mg on D8 and 30 mg on D9), while the CRS was only grade 2 with short period of high fever. Pt #16 showed persistent fever (40 days) with the complication of lung infection during treatment, and therefore was treated with low doses of dexamethasone (10 mg daily form D10 to D14) due to considerations of high level of serum Ferritin and pleural effusion which was already present before CART treatment. Pt #18 experienced severe cardiotoxicity during CRS, which was successfully rescued after administration of dexamethasone (10 mg every 6 h on D8) and methylprednisolone (120 mg every 12 h from D9 to D16).

### Clinical efficacy was not compromised in CART-secreting aIL6 and IL1RA

It is encouraging that our results of initial 18 patients clearly demonstrated that CART-secreted aIL6 scFv and IL1RA could efficiently restrain IL6 and IL1 elevation during CRS, therefore minimizing IL6- and IL1-associated toxicity during CART therapy. Equally important is that the efficacy of clinical response was not affected by the incorporation of IL6 and IL1 blockers. Firstly, the rationale design in this study is engineering CART to coexpress aIL6 scFv and IL1RA and maintaining the essential intercellular signaling domains of CD3ζ and 41BB within the CAR structure. Secondly, there has been no clinical study suggesting that IL6 signaling blockade by Tocilizumab would impair CART therapeutic efficacy^16^. Furthermore, there has been no evidence showing that IL1 signaling is critical for CART-mediated anti-tumor functions.

**Fig. 5:**
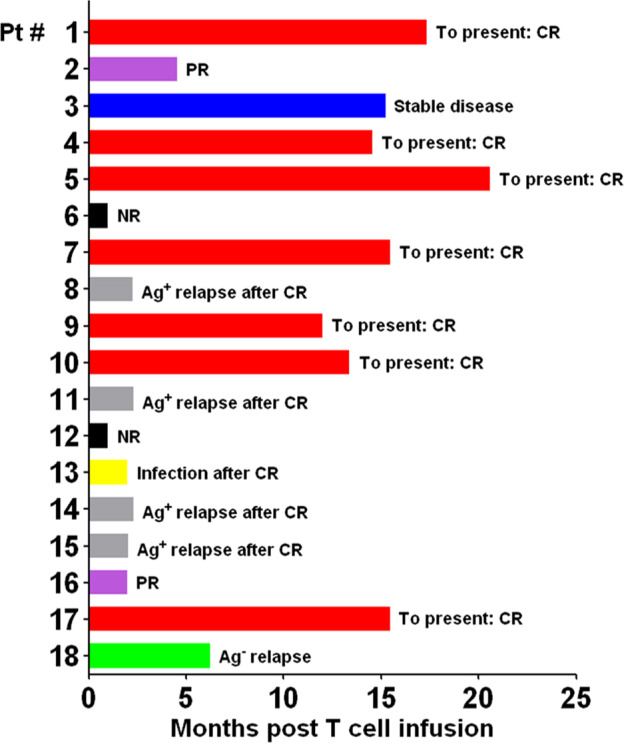
Long term survival of patients (as numbered in Table 1) treated with CART-aIL6/IL1RA. Patients with refractory or relapsed ALL, CLL, Lymphoma or MM were enrolled and treated with anti-CD19 or anti-BCMA CART secreting anti-IL6 scFv and IL1RA. After CART infusion, patients were examined for clinical response of tumor remission, and followed up for evaluating long-term survival. CR complete response, PR partial response, NR no response, Ag^+^ relapse antigen positive relapse, Ag^–^ relapse antigen negative relapse.

In the current study, 90% (9/10) patients with ALL, 40% (2/5) with Lymphoma, and 100% (2/2) patients with MM achieved CR, while 40% (2/5) with Lymphoma achieved PR as shown in Table 1. Although the Pt #16 with CLL showed significant CART expansion and tumor eradication in bone marrow, PR was achieved in the primary tumor site around neck because the tumor was huge sized and highly rigid as solid tumor. Similarly, Pt #12 showed efficient CART expansion, but no response was observed in the tumor site around spinal cord. Pt #2 and #3 showed limited expansion and achieved only partial response. In the patients with CR, our CART showed significant expansion after infusion (Fig. 2a). More importantly, there are 3/10 ALL, 3/5 lymphoma and 2/2 MM patients undergoing progression-free survival for more than 12 months (Fig. 5). In sum, high rate of CR and significant CART expansion in treated patients strongly substantiated that CART-aIL6/IL1RA is not compromised in regard to clinical therapeutic efficacy.

### High level of serum IL6 alone caused neurotoxicity after CRS ended

**Fig. 6:**
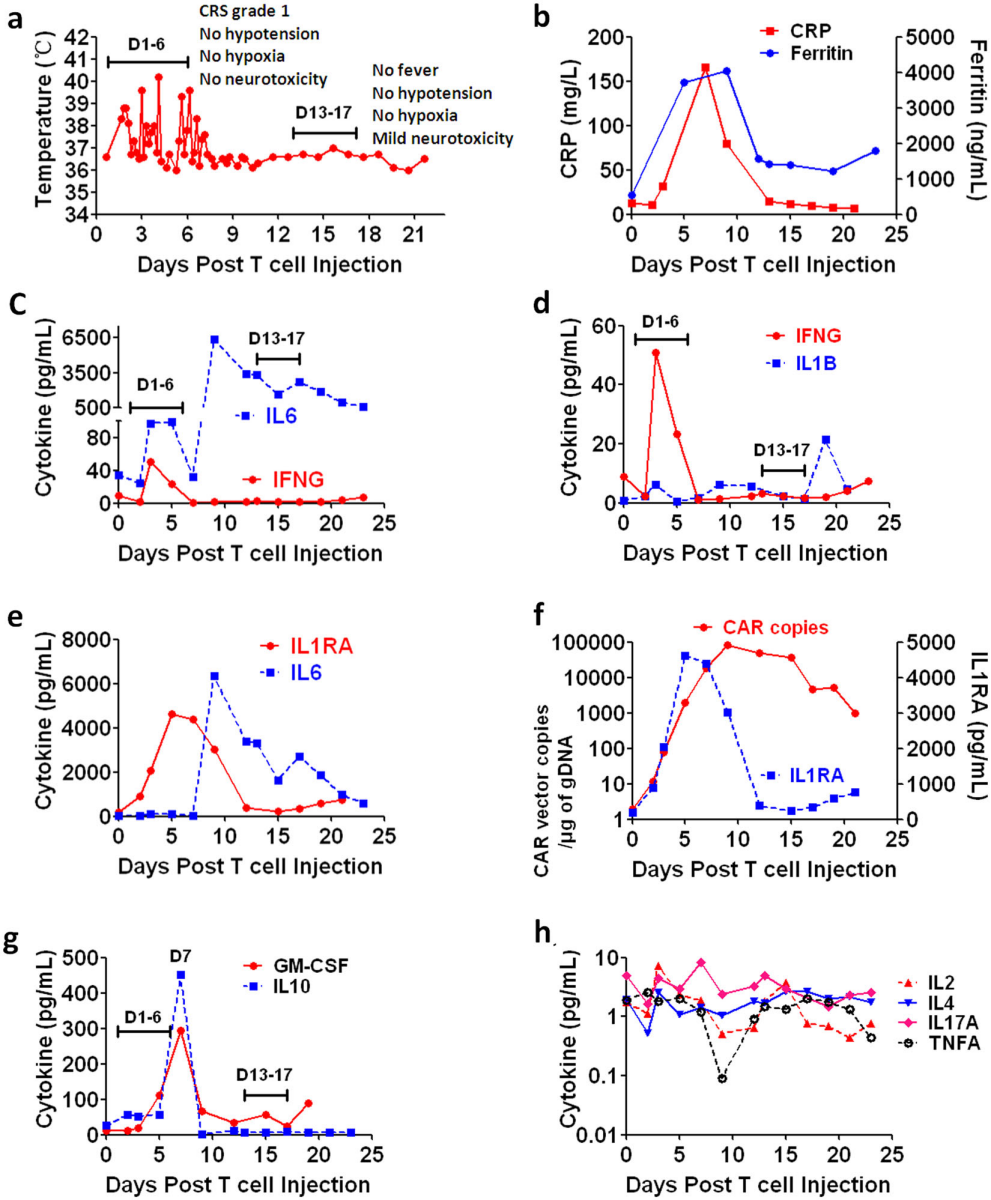
Sustained high level of serum IL6 was associated with neurotoxicity in the patient (#1 in Table 1) after typical grade 1 CRS ended. **a** The changes of body temperature after infusion. **b** Levels of CRP and Ferritin in peripheral blood. **c** Changes of IFNG versus IL6. **d** Changes of IFNG vs IL1B. **e** Changes of IL1RA vs IL6. **f** Kinetics of CAR vector copies vs IL1RA changes. **g** Changes of GM-CSF and IL10. **h** Changes of IL2, IL4, IL17A, and TNFA.

Interestingly, there was only one patient (Pt #1) showing Grade 1 CRS with fever from D1 to D6 (without hypoxia, hypotension, or neurotoxicity), and neurotoxicity from D13 to D17 after CRS ended (Fig. 6a). Consistently, levels of CRP and Ferritin peaked at D7 and decreased to low levels during D13–17 (Fig. 6b). Analysis of cytokines revealed low levels of IFNG and IL6 during D1 to D6 (Fig. 6c). However, IL6 increased dramatically at D9 as IFNG decreased to baseline levels and was maintained at high levels from D9 to D23 (Fig. 6c). IL1B was maintained at very low levels throughout the treatment (Fig. 6d). As shown in Fig. 6e, we observed a reverse correlation between IL1RA and IL6 levels, suggesting that IL6 was effectively neutralized by CART co-secreted aIL6 scFv during CRS and significantly increased due to decrease of CART co-secreted aIL6 scFv after CRS ended. Consistently IL1RA increased significantly as CART expanded and decreased gradually after CRS ended (Fig. 6f), suggesting a similar trend of co-expressed aIL6 scFv. Analysis of additional cytokines revealed moderate levels of GM-CSF and IL10 peaking at D7 (Fig. 6g), and very low levels of IL2, IL4, IL17A, and TNFA (Fig. 6h), all of which were maintained at low levels during D13 to D17. These results together suggested that high level of serum IL6 alone caused neurotoxicity. The mechanism underlying this interesting phenomenon might be attributed to that macrophages remained activated to synthesize IL6 after tumor eradication, whereas CART-secreted anti-IL6 scFv significantly decreased, leading to significant IL6 elevation. Coincidently, this patient showed renal dysfunction and therefore could not clear the high level of serum IL6 even after CRS ended, which led to sustained high level of serum IL6 to cross BBB for inducing neurotoxicity. These observations provide direct evidence that high level of serum IL6 alone could cause neurotoxicity and that direct neutralization of serum IL6 during CRS serves as a desirable strategy for minimizing IL6-associated cytokine toxicity and neurotoxicity.

### Adverse events observed in patients treated with CART-aIL6/IL1RA

All adverse events (AE) during treatment were summarized in Table 2. Certain AE present in more than 50% patients include pyrexia, increased CRP and Ferritin, hypotension, tachycardia, neutropenia, thrombocytiopenia, anemia, and elevated D-dimer(> 0.55). The cause of pyrexia, increased CRP and Ferritin, hypotension and tachycardia could be attributed to CRS. Neutropenia, thrombocytiopenia and anemia were usually caused by preconditioning of thermotherapy for reducing tumor burden and lymphodepletion. While neutropenia-associated infection has been reported as a common AE during CART-cell therapy in 10%–20% of patients^3^, we also observed 5 patients (#10, 11, 13, 16, and 18) showing infections during treatment associated with typical symptoms of neutropenia, thrombocytiopenia, and anemia. In particular for Pt #13, significantly elevated level of baseline IL6 implied the presence of infection right before CART infusion. In Pt #16 showing lung infection, elevated levels of IL6 comparable to baseline were observed throughout the treatment, suggesting that CART-secreted aIL6 did not completely neutralize IL6. Meanwhile, it is important to note that IL1RA elevation in all patients showed a transient pattern during treatment (Fig. 2a), which was correlated with the peak of CART expansion and suggested that CART-secreted aIL6 would be expected to be transient as well. Therefore, transient expression of CART-secreted aIL6 and IL1RA could minimize potential risk of increasing possibility of infection during CART treatment by impairing patients’ immunity through IL6 and IL1 sigaling blockade. In summary, we observed CRS-associated AE, preconditioning caused neutropenia and associated infections, which were commonly present in CART treatments and therefore supported safety of CART-aIL6/IL1RA in clinical application.

**Table 2:** Adverse events (AE) in treated patients.

Patient#	1	2	3	4	5	6	7	8	9	10	11	12	13	14	15	16	17	18	Sum (%)
*General disorders*																			
Pyrexia (≥38 °C)	√		√		√	√	√	√	√	√	√	√	√	√	√	√	√	√	89
Malaise/fatigue	√										√								11
Myalgias																			0
Arthralgias																			0
Edema																		√	6
Headache	√										√								11
Dizziness																			0
Rigor																			0
*Laboratory tests*																			
Increased CRP(>8 mg/L)	√		√		√	√		√	√	√	√	√	√	√	√	√	√	√	89
Increased SF (Female >150 ng/mL, Male >200 ng/mL)	√	√	√		√	√		√	√	√	√	√		√	√	√	√	√	89
*Cardiovascular system*																			
Hypotension																			
No vasopressor			√				√				√		√	√					28
Vasopressor															√	√	√	√	28
Arrhythmia																			
Tachycardia	√		√					√		√			√	√	√			√	44
Bradycardia						√					√								11
Pemature beat															√				6
QT prolongation, ST-T changes																		√	6
Decreased LVEF																		√	6
Pericardial effusion											√			√		√		√	22
Elevated troponin																			0
*Respiratory system*																			
Dyspnea						√									√	√		√	22
Hypoxemia													√			√		√	17
Ventilator support																			0
Pleural effusion									√		√					√		√	22
*Renal system*																			
Increased creatinine	√																		6
Gastrointestinal system																			
Nausea/vomitting										√	√			√					17
Diarrhea					√				√	√				√				√	22
Abdominal pain									√										6
Edema of the pancreas or gallbladder																		√	6
Elevated liver enzymes																			
Increased alanine aminotransferase										0	2						1	0	11
Increased aspartate aminotransferase										2	4						3	3	22
ALP								3	1			1		1	1	0	1	2	33
Increased total bilirubin												2							6
Hematologic toxicity																			
Neutropenia																			
Mild (<1500/µL)		√	√		√		√												22
Severe (<500/µL)	√					√		√	√	√	√	√	√	√	√	√	√		67
Thrombocytiopenia																			
Mild (<150 × 10^3^/µL)		√			√		√			√					√				28
Severe (<50 × 10^3^/µL)	√					√		√	√		√	√	√	√		√	√	√	61
Anemia																			
Mild to moderate anemia(≥60 g/L)	√				√	√	√		√	√	√	√					√		50
Severe anemia (<60 g/L)								√						√	√	√		√	28
*Coagulation*																			
Elevated D-dimer > 0.55								√	√	√	√	√	√	√	√	√	√	√	61
Hypofibrinogenemia ± bleeding <1.7											√					√		√	17
Prolonged PT									√	√	√							√	22
Prolonged APTT								√	√	√	√							√	22
*Neurologic events*																			
Delirium	√																		6
Convulsion																			0
Anxiety																			0
Somnolence																			0
Tremor	√																		6
Disturbance in attention																			0
Speech disorder																			0
Abnormal thinking																			0
*Infections*																			
										√	√		√			√		√	28

Certain AE was graded according to NCI CTCAE.

## Discussion

In this study, we demonstrated that CART-secreted cytokine antagonists could self-neutralize IL6 storm and block IL1 signaling. Our results indicated that treated patients experienced reduced cytokine toxicity and minimal neurotoxicity associated with IL6 and IL1. However, there were still 4 out 18 patients showing grade 3 CRS, which might be attributed to tumor lysis syndrome and other cytokines that were significantly elevated during CART therapy, which might involve GM-CSF, G-CSF, MCP-1, TNFA, MIP-1, IL8, IL13, and IL10. Further investigation is still needed to identify whether additional cytokines are involved in causing severe CRS and the source of such cytokines, which will allow scientists to optimize the CART design for further reducing cytokine toxicity. Furthermore, our results suggested that CART proliferation and anti-tumor efficacy were not impaired in treated patients.

In contrast to Tocilizumab binding to IL6R, our CART-secreted anti-IL6 scFv directly targets and neutralizes IL6, which will minimize the possibility of serum IL6 to cross BBB for causing potential neurotoxicity. Compared to full-sized antibody Tocilizumab, small sized anti-IL6 scFv possesses much better penetration capacity and shorter half-life, which will allow efficient neutralization of systemic IL6 and relieve the safety concern of long lasting IL6 blockade in compromising patient immunity. Efficient neutralization of IL6 by CART-secreted anti-IL6 scFv supports the idea of applying FDA-approved anti-IL6 monoclonal antibody for rescuing IL6 toxicity during CART therapy, such as Siltuximab, which might be a valuable alternative to Tocilizumab in blocking extremely high level of IL6 storm.

As anti-CD19 CART therapy has been approved in clinics, latest anti-BCMA CART therapy has shown promising clinical effects and is close to FDA approval. Our study here demonstrated proof of concept that automatic cytokine blockade could be achieved by CART-secreted cytokine antagonists. In regard to hematologic malignancy, wide distribution of tumor cells and antigen-positive normal cells is the intrinsic driving force to significant CART expansion and tumor lysis to potentially induce severe cytokine toxicity. Therefore, our automatic IL6/IL1 blockade platform serves as a promising strategy to minimize IL6/IL1-associated cytokine toxicity and neurotoxiticy for future development of new target directed CART therapy against hematologic malignancy, or gene edited production of off-shelf allogeneic CART cells.

## Materials and methods

### Patients enrollment

The clinical studies had been approved by the ethics committee of clinical trials in The First Affiliated Hospital of USTC and The Second Xiangya Hospital of Central South University, and registered on Chinese Clinical Trial Registry with accession numbers ChiCTR2000032124 and ChiCTR2000031868. In brief, patients with refractory or relapsed ALL, CLL, Lymphoma, or MM were enrolled and peripheral blood was collected for CART ex vivo production. CAR was composed of the second generation 41BBζ signaling and extracellular antigen recognizing scFv derived from FMC63^24^ or C11D5.3^25^ targeting human CD19 or BCMA. Before infusion, patients were pretreated with lymphodepletion regimen Fludarabine/Cyclophosphamide (F/C). After infusion with CART-aIL6/IL1RA (anti-CD19 CART for ALL, CLL, Lymphoma, and anti-BCMA CART for MM), patients were monitored for clinical signs of CRS and examined for clinical response of tumor remission. CRS was graded according to the ASTCT consensus criteria as referenced^22^.

### Assessment of clinical response

(1) For patient with ALL, bone marrow examination was performed within 1 month after CD19 CART cells infusion to detect morphology, and MRD by multicolor flow cytometry or further tested for mutant gene markers by quantitative polymerase chain reaction (qPCR) if applicable. B cell frequency in peripheral blood was also tested to confirm the clinical response. CR is defined as less than 0.01% marrow blasts by flow cytometry or mutant gene markers by qPCR.

(2) For patients with Lymphoma/CLL, patients were monitored according to standard of care with positron emission tomography (PET) before lympho-depletion chemotherapy and ~4–8 weeks after CART cell infusion, as clinically indicated. Bone marrow aspiration and biopsy were obtained before lymphodepletion and ~4 weeks after administration of CART cells from patients with marrow disease on initial staging, and as clinically indicated thereafter. Best responses in the absence of additional anti-tumor therapy were reported according to the Lugano criteria.

(3) For patient with MM, clinical response and disease progression were assessed within 1–2 month after BCMA CART cells infusion according to the International Myeloma Working Group (IMWG) based on the M-protein level in serum and urine indicating paraproteinemia and paraproteinuria. Then minimal residual disease (MRD) in BM was detected by eight-color flow cytometry with a limit of detection of 0.01%.

### Identification of scFv for blocking IL6 signaling

HEK293T cells were transfected with 3rd generation self-inactivating (SIN) lentiviral transfer vectors encoding scFv antibody derived from monoclonal antibodies targeting IL6 or IL6R, by Lipofectamine 2000 (Thermo Scientific). The supernatants of transfected cells, containing the scFv antibodies expressed by the transfected HEK293T cells, were collected, diluted, and added to HEK-Blue IL6 reporter cells (Invivogen) in the presence of 2 ng/mL human IL6. HEK-Blue IL6 reporter cells were used because they are capable of producing secreted embryonic alkaline phosphatase (SEAP) upon human IL6 stimulation. After overnight incubation, the supernatant of HEK-Blue IL6 cells was collected and incubated with Quant-Blue substrate solution. SEAP production was quantified by measuring optical absorbance of converted substrate Quant Blue (Invivogen) at 650 nm wavelength through a spectrophotometer.

### Co-expression of aIL6 scFv and IL1RA

Nucleic acids encoding different formats of IL1RA with aIL6 scFv were cloned into the 3rd generation self-inactivating (SIN) lentiviral transfer vector. 293T cells were transfected with a lentiviral transfer vector as described above by Lipofectamine 2000 (Thermo Scientific). The supernatant from the transfected cells was collected and added to HEK-Blue IL1R Cells (Invivogen) in different dilutions as indicated in the presence of 10 pg/mL IL1B. After overnight incubation, the supernatant of HEK-Blue IL-1R Cells was collected and incubated with substrate solution of Quant-Blue (Invivogen), and the optical absorbance of converted substrate was measured at 650 nm wavelength through a spectrophotometer. The supernatant was also added to HEK-Blue IL6 Cells (Invivogen) in different dilutions as indicated in the presence of 2 ng/mL IL6. After overnight incubation, the supernatant of HEK-Blue IL6 Cells was collected and incubated with substrate solution of Quant-Blue (Invivogen), and the optical absorbance of converted substrate was measured at 650 nm wavelength through a spectrophotometer.

### CART cells ex vivo expansion

Peripheral blood was collected from the enrolled patients and processed with Ficoll (GE Healthcare) gradient centrifugation to isolate PBMC. T cells were then purified from PBMC with the pan T isolation kit (Miltenyi). Purified T cells were activated with anti-CD3&CD28 dynabeads (Thermo Fisher) and transduced with 3rd generation lenti-vector encoding anti-CD19 or anti-BCMA CAR and IL6/IL1 blockers. T cells were further expanded and analyzed for CAR expression (Novocyte, Agilent). The CART cells were also tested for sterility and in vitro killing of leukemia cells Nalm6 or RPMI 8226 cells (ATCC) expressing GFP (Novocyte, Agilent).

### Flowcytometry analysis of CAR expression

CAR was stained by a primary biotinlated goat-anti-mouse Fab antibody and secondary PE-Strepavidin (Jackson Immune), followed by analysis on Novocyte (Agilent).

### Analysis of cytokines by ELISA

The peripheral blood samples were collected from infused patients. The plasma samples were analyzed by ELISA kits (Boster) according to the manufacturer’s protocol to measure the concentrations of cytokines after CART infusion.

### qPCR analysis of CAR vector copies

Genomic DNA was purified from patient blood sample by gDNA isolation kit (Thermo Fisher), and analyzed with SYBR Green qPCR kit (Takara) according to manufacturer’s protocol on Lightcycler (Roche).

### Animal study

Animal study was approved by IACUC committee in Department of Laboratory Animals of Central South University. 6–8-week old NCG mice (purchased from GemPharmatech) were intravenously injected with 1 × 10^6^ Nalm6 leukemia cells modified to stably express GFP. 7 days later, the mice were intravenously injected with 2 × 10^6^ anti-CD19 CART cells, which also express aIL6 fused with human IgG Fc (indicated as “CART-aIL6/Fc” in the figures, *n* = 8) or human IgG Fc only (indicated as “CART-Fc” in the figures, *n* = 7). Mice not receiving CART cells were included as control (indicated as “CTRL” in the figures, *n* = 5). Post T cells injection, the mice were monitored for body weight, survival, and the number of CD19^+^GFP^+^ Nalm6 leukemia cells and CD45^+^CD3^–^ T cells in the blood by Novoexpress (Agilent). CART-secreted aIL6/Fc was analyzed by human IgG ELISA kit from Abcam.

### Statistical analysis

The Student’s *t*-test was conducted to evaluate statistical significance. *Denotes statistical significance with *P* < 0.05. ns means no significance.
